# Experiences of secondary health conditions amongst people with spinal cord injury in South Africa: A qualitative study

**DOI:** 10.4102/sajp.v77i1.1530

**Published:** 2021-04-06

**Authors:** Sonti I. Pilusa, Hellen Myezwa, Joanne Potterton

**Affiliations:** 1 Department of Physiotherapy, School of Therapeutic Sciences, Faculty of Health Sciences, University of the Witwatersrand, Parktown, South Africa

**Keywords:** secondary health conditions, secondary complications, spinal cord injury, health, wellbeing

## Abstract

**Background:**

Secondary health conditions (SHCs) such as pain, pressure sores, sexual problems, bowel and bladder problems are prevalent throughout the lifespan of people with spinal cord injury (SCI). Studies have reported that SHCs decrease life expectancy and increase health care costs. Studies on the lived experience of SHCs are, however, limited.

**Objectives:**

To explore the experiences of SHCs amongst people with SCI in a public rehabilitation hospital in South Africa.

**Method:**

Face-to-face semi-structured interviews were conducted with people with SCI from August 2018 to July 2019. All interviews were transcribed verbatim and analysed using a content analysis approach.

**Results:**

Seventeen people with SCI were interviewed. Participants experienced a range of SHCs. The most common experienced SHC was pain (94%). The main theme that emerged from the analysis was ‘the impact of secondary health conditions on health and well-being’. The categories linked to the impact were SHCs co-occurrence and how SHCs limit function, restrict participation, affect mental health and disrupt lives.

**Conclusion:**

We found that SHCs were enormously impactful on our participants’ lives and health, as illustrated by their stories of fear, embarrassment and shame. Understanding people with SCI experiences of SHCs can enhance communication between people with SCI and health professionals and may help develop prevention strategies.

**Clinical implications:**

To enhance patient-centred care, health professionals are encouraged to actively listen to patients’ experiences of illness and the impact on health and wellbeing.

## Background

Health is a complex phenomenon that extends beyond dysfunction or impairments. It is a by-product of the interaction between the affected bodily structures and systems, and ability to function and participate in life within a specific context (World Health Organization 2013). Health incorporates a sense of positive emotions about self, the ability to adapt, self-manage and thrive (Huber et al. 2011). Spinal cord injury (SCI) affects physical, mental and social health domains (Fuseini, Aniteye & Alhassan 2019; Jörgensen, Iwarsson & Lexell 2017). Furthermore, across the lifespan, people with SCI are at risk of developing secondary health conditions (SHCs), which lead to poor health (Mittmann, Hitzig & Craven 2014) and quality of life (Mashola & Mothabeng 2019).

Secondary health conditions are health problems, not necessarily caused by the SCI, but which occur because of living with a disability (Rimmer, Chen & Hsieh 2011). The different SHCs that may develop after SCI include pain (neuropathic and musculoskeletal), pressure sores, spasms, postural hypotension, pneumonia, bowel and bladder problems, mental health problems and sexual problems (Brinkhof et al. 2016; Jensen et al. 2013). There is evidence that SHCs are prevalent throughout the lifespan of people with SCI. Some SHCs become more prevalent over time (such as urinary tract infections), whilst others may increase in severity (such as musculoskeletal pain) (Adriaansen et al. 2013, 2016). Certain SHCs remain constant (such as constipation and pressure sores) whilst others may reduce (bowel incontinence and spasticity) over time (Adriaansen et al. 2016). The reported SHCs prevalence in South Africa during the acute hospital phase was estimated at 50% (Joseph & Wikmar 2016), which is markedly lower than that reported in Sweden (78%) (Wahman et al. 2019). The different estimates could be because of the participants having different SCI profiles. Post rehabilitation estimates of SHCs are higher: 89% in South Africa (Madasa et al. 2020) and 98.5% in the Netherlands (Adriaansen et al. 2016). Given the high prevalence of SHCs post-rehabilitation, it is worth exploring how people with SCI experience SHCs.

In South Africa, studies on SHCs in people with SCI are limited and only three studies have reported on the occurrence of SHCs in the post-rehabilitation phase (Madasa et al. 2020; Mashola & Mothabeng 2019; Mashola, Olorunju & Mothabeng 2019). These studies do not explore the experience of living with SHCs. Madasa et al. (2020) reported the mortality rate and the prevalence of SHCs amongst survivors at 4 years post-injury. Mashola et al. (2019) reported on the high hospital readmission rates of patients with SCI because of SHCs and Mashola and Mothabeng (2019) on the impact of SHCs on quality of life. The latter studies were conducted in a private rehabilitation facility. Thus, there is still a lack of knowledge on the SHCs experienced by people with SCI in public health facilities. Evidence on SHCs experienced by people with SCI in the public health care system is essential because the majority (84%) of South Africans do not have access to health insurance and depend on the already strained public health system (Mayosi & Benatar 2014). There is a need for more, contextually specific, qualitative studies on patients’ narratives of their experiences of SHCs. Understanding patients’ ill-health experiences can inform the context-based prevention care model of care for people with SCI. Our research question is: *what are the experiences of SHCs, and how do SHCs affect individuals with SCI*? Thus, the objective of our study was to explore the experiences of SHCs amongst people with SCI.

## Method

This study is part of a more extensive study that aims to establish factors influencing prevention of SHCs in people with SCI to inform the development of a preventative model of care. We used a phenomenological qualitative approach to give clarity and a more in-depth understanding of the experiences of SHCs (Austin & Sutton 2014; Neubauer, Witkop & Varpio 2019). Relying on the interpretivist paradigm, semi-structured interviews were conducted to explore and gain a deeper understanding of this phenomenon (Thanh, Thi & Thanh 2015).

Our study was based at a 79-bed public tertiary rehabilitation hospital in Gauteng province, South Africa. This hospital also admits patients from other provinces because of the lack of tertiary hospitals in other provinces. The hospital offers inpatient rehabilitation services to patients with stroke, head injuries, SCI and amputation and aims to help patients achieve their highest functional ability through therapy, education programmes, peer support and work-oriented activities. The services available include medical care, pharmacists care, occupational therapy, physiotherapy, speech therapy, dietetics, social work and psychology. Participants who were eligible and consented after the staff approached them were included in our study. A total of 25 people with SCI were approached, 20 of whom were eligible and consented to participate. Two had health-related problems, and one had time constraints thus were not available for the interview. All adult (>18 years) potential participants were considered regardless of gender, time since SCI and injury profile, such as the type of injury and the lesion’s completeness. Patients who presented with other neurological conditions (stroke and head injury) were excluded.

## Data collection

Semi-structured interviews were conducted using an interview guide developed using the literature related to SHCs and preventative care (Guilcher et al. 2013). The interview was piloted with a researcher experienced in qualitative methods to clarify the questions. Part A of the interview guide included questions on sociodemographic information including age, sex, marital status, injury date, cause and the level of the injury, completeness of injury, occupation and use of an assistive device for mobility. Part B of the interview guide included questions on the experiences of SHCs. The first author obtained written informed consent for the interviews and audio recordings. The interviews were then conducted from August 2018 to July 2019, in the participant’s language of choice at the preferred venue and lasted for 45 min on average. Five participants were readmitted for pressure sores, thus were interviewed in the inpatient section of the hospital. When interviews and analysis yielded no new information, data collection was discontinued (Hennink, Kaiser & Marconi 2017).

## Data analysis

The interviews that were in the vernacular were translated into English by an independent translator and translation checked before analysis by the first author. Interviews were transcribed verbatim and exported to MAXQDA version 2018.1: a computer-assisted qualitative data analysis software. We used a content analysis approach to analyse the data and understand the participants’ experiences. This process entails transforming text into meaningful units, codes, categories and themes (Erlingsson & Brysiewicz 2017). The transcripts were read several times to understand the main ideas expressed by the participants. The authors individually coded a transcript inductively, followed by a discussion on the codes and the categories. A coding framework was developed and used to code the rest of the transcripts deductively. Manual categorisation was conducted by the first author and reviewed by the co-authors. A further abstraction of the data to develop themes related to our study objective was performed by all the authors. The following strategies were employed to ensure data trustworthiness. Code recoding was completed, and final categories were compared with the previous analysis. Debriefing sessions amongst the authors were held throughout the research process to discuss the findings and data analysis. To ensure confirmability of the data, all the interviews were audio-recorded, field notes were kept and the first author included a reflexive analysis of the interviews.

Our research experience related to disability has informed the analysis of the data. Having worked as therapists in school settings for children with disabilities and non-governmental organisations for people with disabilities, we realise how it is easy to focus on impairments and functional limitations and not look at the whole person to understand the impact of SHCs experienced.

### Ethical considerations

Permission to use the study site for data collection was granted by the rehabilitation hospitals. Our study was approved by the Human Research Ethics Committee of the University of the Witwatersrand (M170938) and it was registered on the South African National Health Research Database (GP201712036).

## Results

### Part A: Demographic profile of the participants

A total of 17 people with SCI were interviewed. The mean age (years) was 44.5 (σ = 13.1) with a range of 27–72 years. The mean time since injury was 9 years (σ = 7.1) and the range was between 1 and 30 years. Table 1 depicts the demographic information collected from Part A of the interview.

**TABLE 1:** Demographic profile of the participants (*n* = 17).

Description	*n*	%
**Gender**		
Male	14	82.4
Female	3	17.6
**Employment**		
Yes	5	29.4
No	12	70.6
**Education level**		
Tertiary education	4	23.5
Matric	5	29.4
High school	6	35.3
Primary school	2	11.8
**Marital status**		
Married or staying with a partner	7	41.2
Single	10	58.8
**Cause of injury**		
Trauma	14	82.4
Non-trauma	3	17.6
**Type of spinal cord injury**		
Paraplegia	14	82.4
Tetraplegia	3	17.6
**Completeness of the injury**		
Incomplete	4	23.5
Complete	13	76.5
**Level of the injury**		
C1–C4	2	11.8
C5–T1	1	5.9
T2–T6	3	17.6
T7–T12	9	52.9
L1–L5	2	11.8
**Assistive device**		
Wheelchair	14	82.3
Walking aid	2	11.8
None	1	5.9

### Part B: Range of secondary health conditions **experienced**

Table 2 depicts the range of SHCs experienced by the participants.

**TABLE 2:** Range of secondary health conditions experienced (*n* = 17).

Type of secondary health condition	*n*	%
Pain	16	94
**Bladder problems**		
Incontinence	16	94
Urinary tract infection	4	24
Spastic bladder	2	12
Kidney stones	1	6
**Bowel problems**		
Incontinence	11	65
Constipation	6	35
Rectal prolapsed	1	6
Bloating	1	6
Psychological problems (depression, worry and stress)	12	71
Pressure sores	10	59
Spasms	9	53
Contractures	3	18
**Injuries**		
Burns	9	53
Fractures	4	24
Falls	8	47
Sexual issues	7	41
Sleeping disturbances	6	35
Fatigue	4	24
**Skeletal problems**		
Osteoporosis	2	12
Arthritis	1	6
Myositis ossificans	1	6
Respiratory problems	1	6

Participants reported a range of SHCs presented in Table 2. The most common SHC was pain (94%).

### Part C: Experience of secondary health conditions

The main theme that emerged from the analysis was the ‘impact of SHCs on health and well-being’. There were five categories linked to the main theme: namely, SHCs co-occurrence, limits function, restricts participation, mental health effects and disrupts lives.

#### Impact of secondary health conditions on health

Secondary health conditions had a negative impact on the different domains of health, body structures and systems, activities and participation in life. Also, the SHCs affected mental well-being and disrupted the usual routine of life.

**Secondary health conditions co-occurrence:** The co-occurrence of SHCs was noted in osteoporotic bones and fractures, spasms and pain. The presence of one SHC increases vulnerability to other SHCs:

‘The cramp is on my legs, it goes hand in hand with pain.’ (Jane, female, 27 years old)

‘I have osteoporosis – I put my hand under my leg, and as I turned over the femur then I broke my femur.’ (Julie, female, 61 years old)

‘I didn’t sleep yesterday because of spasms and pains.’ (Jared, male, 35 years old)

**Limits function:** The presence of SHCs limited functioning and mobility. Participants who experienced pressure sores were required to lie in bed for an extended time and thus developed weakness as reported by Jared:

‘The other pressure sore got worse and even made a hole – you could even see a bone at the back. I was weak and not even able to get out of bed.’ (Jared, male, 35 years old)

Similarly, the severity of pain affected Joy’s ability to use her assistive devices ‘… some days the pain is unbearable, I can’t hold my crutches.’ (Joy, female, 38 years old).

Participants reported how SHCs affected activities of daily living. Julie (female, 61 years old) said her ankle fracture affected bathing ‘I broke my right ankle. I had to wear a moon boot for eight weeks. It was such a struggle to bath and shower, it was terrible.’ Also, the presence of spasms affected sitting in a wheelchair and made toileting difficult for Joe (male, 38 years old), ‘spasms trouble me when I want to go to the toilet. I can’t sit, when I try to sit, the one leg goes there.’ To prevent further injury because of spasms, one participant reported how he had to rely on medication, ‘I live on Lioresal and Baclofen for spasms. I have to otherwise I don’t stay in the wheelchair’ (Jake, male, 36 years old). Furthermore, Jack highlighted how depression affected self-management and help-seeking behaviour; unfortunately, this made individuals with SCI isolate themselves. ‘Some people get too depressed to a point where they stop coming to get their medication, they just stay at home.’ (Jack, male, 39 years old).

**Restricts participation:** Participants reported how SHCs affected their social lives, occupations and relationships. Secondary health conditions restricted the social lives of the participants. Bowel problems made it challenging for some participants to leave their homes. Some participants were afraid that if they went out somewhere, bowel leaks might happen as expressed by Jared and Jane here:

‘When I have diarrhoea, I don’t leave the house, I just sit, and I use the linen saver that they gave us.’ (Jared, male, 35 years old)

‘What stresses me the most is, what if I go somewhere and number 2 happens? I can prevent number 1, but number 2!’ (Jane, female, 27 years old)

Pain, bladder and bowel problems had a negative impact on work life. Pain affected the participants’ mood at work:

‘The issue of pain when you at work you won’t be happy when you have pain … even if people make jokes when the pain comes you won’t be happy. It stresses me.’ (Jane, female, 27 years old)

Jude had to restrict water consumption when travelling for work-related activities for fear of bladder leakages:

‘I work in a consulting company. Almost every week I go to Johannesburg … I don’t drink a lot of water because if I drink water, I am going to struggle, I am going to leak.’ (Jude, male, 40 years old)

**Affects mental health:** Secondary health conditions affected the mental health of the participants with regard to their emotional and psychological well-being.

**Emotional effects:** The participants felt different emotions related to SHCs such as fear, shame, worry and sadness. Many participants mentioned their fear of bowel leakage in public and the embarrassment of not even noticing the leakage when it occurs:

‘You see, sometimes you can’t feel whether you went for number 1 or 2, maybe you hear it from other people, that’s where the embarrassment comes from; other people have noticed when you haven’t noticed it first.’ (Job, male, 44 years old)

*‘*Number 2 (managing my bowel) troubles my mind … what if I mess on myself? What if there is a smell? Sometimes when I am sitting, I would think there is a smell when I go to the bathroom to check, there is nothing. I become afraid. What if it happens? It’s all in my mind.’ (Jane, female, 27 years old)

Participants reported fear related to pressure sore management. Joe highlighted his fear of dying from a pressure sore that was so severe that he asked to be admitted to a hospital so the health professionals could treat the pressure sore appropriately:

‘I quickly came to the hospital when the pressure sore started because I had a fear that it might kill me, I asked them to admit me because I won’t be able to dress the wound from home.’ (Joe, male, 38 years old)

Pain was a source of worry stated by Job:

‘You see what I like about myself, is that I have accepted that I’m injured. I’m no longer worried by the fact that I cannot walk. The only thing that worries me is the pain – not the inability to walk.’ (Job, male, 44 years old)

Some participants spoke about feelings of sadness and suicidal ideations because of pain as expressed by Julie and Jake:

‘… Pain is the reason why I was always thinking about suicide, it is that pain.’ (Julie, female, 61 years old)

‘… So, that’s where I’ve got most of my pain, every day. I don’t … drink painkillers for it, not normally. Ehmmm … maybe once or … in two months, about. Then…I sit here, and I want to die because then my back is [*fucked*] … sorry … it hurts.’ (Jake, male, 36 years old)

Male participants expressed stress related to sexual problems. They reported how sexual problems challenged their view of manhood, and there was a sense of hopelessness because they had not found a working solution:

‘… Your wife can leave you because you can no longer have sex with her … That is the biggest problem about spinal cord injury. Your partner can still leave you … This can affect you mentally.’ (Jasper, male, 44 years old)

‘Not getting an erection stresses me and I have no solution.’ (Jared, male, 35 years old)

**Psychological effects:** Experiencing SHCs strengthened some participants’ psychological wellbeing. Certain participants found the whole experience of SHCs to be positive in terms of lessons learnt. Valuable lessons learnt included coping strategies, enhancing problem-solving skills, learning new ways of ‘doing things’ and being prepared:

‘I fell out of my wheelchair, and I learnt a valuable lesson that day you must always keep your cell phone with you if you are disabled.’ (Julie, female, 61 years old)

‘[*W*]hen you fall asleep, it feels like somebody is just giving you a pull in your foot. So, you can’t sleep, but you learn to cope with it.’ (Jex, male, 50 years old )

‘I’ve burnt my feet once, so, what am I going to do differently?, do this, do that. I mean, that is something you’ve got to figure out yourself. There is no manual in figuring how to do things differently not to burn the feet again.’ (Jake, male, 36 years old)

**Disrupts lives:** Secondary health conditions disrupted the usual routine of life. Participants expressed that they experienced frequent pressure sores, which required prolonged bed rest and hospital readmission disrupting everyday routine such as going to work:

‘Once it is open, forget it, it will not heal – pressure sores take forever to heal. Mine has been there for eight years.’ (Job, male, 44 years old)

‘You find yourself being bedridden for 6, 7 months because of a pressure sore, it takes you back. Lying on the bed is not nice.’ (Jane, female, 27 years old)

## Discussion

As reported in SCI epidemiological studies, most of the participants were male and had traumatic SCI (Joseph et al. 2015; Sothmann et al. 2015). Unsurprisingly, most participants were unemployed with low education levels, as is often the case for the majority of people with disabilities in South Africa (Hanass-Hancock & McKenzie 2017). Unemployment amongst people with disabilities increases economic vulnerability, which is compounded by out of pocket disability-related costs such as the need for support, accessing health care services and participation in life (Hanass-Hancock & McKenzie 2017; Hanass-Hancock et al. 2017).

The participants experienced a range of SHCs, but the most prevalent SHC was pain (94%). Pain is the most pervasive and significant problem throughout a person’s lifespan with SCI (Madasa et al. 2020; Piatt et al. 2016). When patients report pain symptoms their focus is not on the severity and the location of the pain but the different facets of life affected by pain, namely mental health, physiological impairments and personal behaviour (Müller et al. 2017; Yorkston et al. 2010). Pain affects quality of life (Callaway et al. 2015; Mashola & Mothabeng 2019), sleep and mental health (Turk et al. 2016). To cope with pain, an individual can rely on or misuse medication, avoid certain activities that elicit distress, self-isolate and adopt unhealthy ways to seek attention from their families (Keefe & Gil 1986). Therefore, interventions to manage pain need to be holistic, focusing on helping an individual live well in the presence of pain. Self-management strategies can include stress management, coping strategies and exercise, access to pain medication, counselling and alternative treatment such as massage and continual support.

As in previous studies, we found some of the SHCs (spasms and pain) co-occurred (Brinkhof et al. 2016; Park et al. 2017). Brinkhof et al.’s (2016) survey on health conditions in people with SCI found clustering of sexual dysfunction, spasticity, bowel dysfunction, pain, bladder dysfunction and urinary tract infections (Brinkhof et al. 2016). Albeit, the authors gave no reason for the clustering. Equally, depression and anxiety have been reported to increase the risk of SHCs such as urinary tract infections, pain and spasms (January et al. 2014). Park et al. (2017) found 29% of participants with SCI reported experiencing a combination of sexual dysfunction, bowel and bladder incontinence with a high association between these SHCs. The possible reason for the SHCs co-occurrence could be because of the same parasympathetic innervation to the bowel, bladder and reproductive organs. This evidence shows us that some SHCs co-occur meaning SCI care must include regular screening and comprehensive prevention strategies for known SHCs clusters. More research is still needed to understand other SHCs clustering patterns to inform patient assessment and prevention strategies.

Activities of daily living (ADLs) such as bathing, toileting and mobility were limited by pain and spasms. Callaway et al. (2015) found spasms limited self-care activities such as bathing, transfers and bed mobility. The presence of spasms in a person with SCI is linked to decreased daily living activities and the need for assistance from caregivers (McKay, Sweatman & Field-Fote 2018). Participants also expressed how pain affected them at work. Other studies on pain in people with SCI have reported the negative impact of pain on community integration, including returning to work (Callaway et al. 2015; Donnelly & Eng 2005). Callaway et al.’s (2015) study showed how musculoskeletal pain influenced participants with SCI’s return to work and work choice, with some requiring regular bed rest to relieve pain. We need to support people with SCI to effectively manage and prevent limiting SHCs such as pain and spasms. Support could be in outreach services and home visits by therapists and community-based workers to monitor well-being and functioning at home. Such interventions can promote independence in daily self-care amongst people with SCI.

Participation in social life was affected by bowel and bladder problems and pain. Participants were afraid to leave their homes because of the fear of bladder and bowel leakage. Bowel and bladder problems are problematic SHCs in people with SCI (Piatt et al. 2016), with a prevalence of 44% bladder dysfunction and 42% bowel dysfunction 4 years post-SCI in South Africa (Madasa et al. 2020). A study conducted in Ghana reported that patients with SCI did not want to participate in social events, such as weddings because of bowel and bladder incontinence for fear of leakage in public. The fear of leakage could stem from poor management of both bladder and bowel problems because of inadequate access to consumable such as catheters and a lack of adequate education on bowel and bladder problems (Fuseini et al. 2019). Community participation and integration into the labour market are critical outcomes for rehabilitation care contributing to life satisfaction (Carpenter et al. 2007). Thus, SCI care must incorporate social integration and work rehabilitation.

Mental health is an essential component of health, which includes psychological and mental well-being. This is the awareness of self, personal abilities, stress, coping strategies and the ability to work and contribute meaningfully to society (World Health Organization 2004). Secondary health conditions affected the emotional and psychological well-being of our participants. The presence of SHCs, such as pain, created feelings of sadness and suicidal ideations. Other studies have reported a significant association between pain and depression (Jörgensen et al. 2017a; Robinson-Whelen et al. 2014). Muller’s et al. (2017) study reported that the leading cause for depression in an individual with pain was feeling restricted in participating in life events because of high pain intensity. Therefore, SHCs prevention strategies for pain must include meaningful participation in life activities such as work, community events, leisure and exercise activities.

Furthermore, male participants expressed feelings of stress and hopelessness regarding sexual problems. Sexual issues are the most significant SHCs amongst men, affecting self-identity, relationships and yet they receive the least treatment (Brinkhof et al. 2016; Callaway et al. 2015). Also with regard to mental health, it was interesting to hear participants reporting that the experience of SHCs taught them valuable lessons such as problem-solving skills, coping strategies and lessons to prepare for future incidents. Some participants’ experiences of SHCs developed resilience, a necessary trait for people with chronic conditions, enabling them to adapt and cope with life challenges (Cal et al. 2015; Rohn, Nevedal & Tate 2020). When communicating with people with SCI on challenging experiences of SHCs, it is essential to highlight lessons learnt on problem-solving, coping mechanism because these are positive traits that will help them manage future challenges.

Spinal cord injury is a life-disrupting event affecting personal life and family relationships (Ide-Okochi et al. 2013). Like other studies, we found that SHCs can also disrupt the usual way of living (Fuseini et al. 2019; Nevedal, Kratz & Tate 2016). For example, experiencing pressure sores, lying in one position and being confined to bed for months affects daily routines, separates people with SCI from the outside world and harms their view of self, life and family. As people with SCI are vulnerable to frequent SHCs in their lifespan, we must be more proactive to prevent their occurrence and when they do occur to offer support to patients and the family to manage the disruptions.

In the context of South Africa, our findings provide more evidence to strengthen the use of the biopsychosocial model of care that promotes patient-centred care. The biopsychosocial model of care is holistic and links contextual factors of the patient to care and recognises the patient’s role in care. Patient-centred care is an empowering approach to patient care that encourages the patient’s active participation in relating problems as they experience them, goal setting and decision-making (Lindberg et al. 2013). We need to recognise that the patients’ views on illness and health are shaped by how they experienced them (Kleinman, Eisenberg & Good 1978). Patients want to be listened to and validated by health professionals regarding their health and well-being (Yorkston et al. 2010).

## Conclusion

We explored the experience of SHCs amongst people with SCI and found that SHCs were enormously impactful on people with SCI as illustrated by their stories of fear, embarrassment and shame. Secondary health conditions have a profound impact on the health and well-being of people with SCI. It is not only the presence of SHCs that affect people with SCI but the effect they have on mental health, the ability to function, participate in life and how lives are disrupted. Understanding the experience of SHCs can enhance communication between patients with SCI and health professionals and aid in setting appropriate management and prevention goals.

### Recommendations

We need more studies on the prevention of SHCs and how to minimise their impact on health and well-being. Health providers should be encouraged to engage and actively listen to patients. We encourage clinicians to use open-ended questions during patient assessments, goal setting and treatment to understand how patients experience illness and its impact on their health.

### Limitations

Firstly, given that our study used a qualitative approach, we cannot generalise the findings to other contexts. Secondly, we had very few participants with cervical lesions and female patients with SCI.

## References

[CIT0001] Adriaansen, J.J.E., Post, M.W.M., De Groot, S., Van Asbeck, F.W.A., Stolwijk-Swüste, J.M., Tepper, M. et al., 2013, ‘Secondary health conditions in persons with spinal cord injury: A longitudinal study from one to five years post-discharge’, *Journal of Rehabilation Medicine* 45(11), 1016–1022. 10.2340/16501977-1207 24096367

[CIT0002] Adriaansen, J., Ruijs, L.E.M., Van Koppenhagen, C.F., Van Asbeck, F.W.A., Snoek, G.J., Van Kuppevelt, D. et al., 2016, ‘Secondary health conditions and quality of life in persons living with spinal cord injury for at least ten years’, *Journal of Rehabilitation Medicine* 48(10), 853–860. 10.2340/16501977-2166 27834436

[CIT0003] Austin, Z. & Sutton, J., 2014, ‘Qualitative research: Getting started’, *Canadian Journal of Hospital Pharmacy* 67(6), 436–440. 10.4212/cjhp.v67i6.1406 PMC4275140 25548401

[CIT0004] Brinkhof, M.W.G., Al-Khodairy, A., Eriks-Hoogland, I., Fekete, C., Hinrichs, T., Hund-Georgiadis, M. et al., 2016, ‘Health conditions in people with spinal cord injury: Contemporary evidence from a population-based community survey in Switzerland’, *Journal of Rehabilitation Medicine* 48(2), 197–209. 10.2340/16501977-2039 26931074

[CIT0005] Cal, S.F., Ribeiro de Sá, L., Glustak, M.E. & Santiago, M.B., 2015, ‘Resilience in chronic diseases: A systematic review’, *Cogent Psychology* 2(1), Article ID 1024928. 10.1080/23311908.2015.1024928

[CIT0006] Callaway, L., Barclay, L., McDonald, R., Farnworth, L. & Casey, J., 2015, ‘Secondary health conditions experienced by people with spinal cord injury within community living: Implications for a National Disability Insurance Scheme’, *Australian Occupational Therapy Journal* 62(4), 246–254. 10.1111/1440-1630.12206 26256853

[CIT0007] Carpenter, C., Forwell, S.J., Jongbloed, L.E. & Backman, C.L., 2007, ‘Community participation after spinal cord injury’, *Archives of Physical Med icine and Rehabilation* 88(4), 427–433. 10.1016/j.apmr.2006.12.043 17398242

[CIT0008] Donnelly, C. & Eng, J.J., 2005, ‘Pain following spinal cord injury: The impact on community reintegration’, *Spinal Cord* 43(5), 278–282. 10.1038/sj.sc.3101702 15570317 PMC3226795

[CIT0009] Erlingsson, C. & Brysiewicz, P., 2017, ‘A hands-on guide to doing content analysis’, *African Journal of Emergency Medicine* 7(3), 93–99. 10.1016/j.afjem.2017.08.001 30456117 PMC6234169

[CIT0010] Fuseini, A., Aniteye, P. & Alhassan, A., 2019, ‘Beyond the diagnosis: Lived experiences of persons with spinal cord injury in a selected town in Ghana’, *Neurology Research International* 2019, 1–10. 10.1155/2019/9695740 PMC6354163 30792925

[CIT0011] Guilcher, S.J., Craven, B.C., Lemieux-Charles, L., Casciaro, T., McColl, M.A. & Jaglal, S.B., 2013, ‘Secondary health conditions and spinal cord injury: An uphill battle in the journey of care’, *Disability and Rehabilitation* 35(11), 894–906. 10.3109/09638288.2012.721048 23020250 PMC3665227

[CIT0012] Hanass-Hancock, J. & McKenzie, T.C., 2017, ‘People with disabilities and income-related social protection measures in South Africa: Where is the gap?’, *African Journal of Disability* 6, 1–11. 10.4102/ajod.v6i0.300 PMC5645570 29062759

[CIT0013] Hanass-Hancock, J., Nene, S., Deghaye, N. & Pillay, S., 2017, ‘“These are not luxuries, it is essential for access to life”: Disability related out-of-pocket costs as a driver of economic vulnerability in South Africa’, *African Journal of Disability* 6, 280. 10.4102/ajod.v6i0.280 28730066 PMC5502471

[CIT0014] Hennink, M.M., Kaiser, B.N. & Marconi, V.C., 2017, ‘Code saturation versus meaning saturation: How many interviews are enough?’, *Qualitative Health Research* 27(4), 591–608. 10.1177/1049732316665344 27670770 PMC9359070

[CIT0015] Huber, M., Knottnerus, J., Lawrence, G., Henriëtte, H., Alejandro, J., Daan, K. et al., 2011, ‘How should we define health?’, *BMJ (Online)* 343(7817), Article ID d4163. 10.1136/bmj.d4163

[CIT0016] Ide-Okochi, A., Yamazaki, Y., Tadaka, E., Fujimura, K. & Kusunaga, T., 2013, ‘Illness experience of adults with cervical spinal cord injury in Japan: A qualitative investigation’, *BMC Public Health* 13(69), 1–10. 23347726 10.1186/1471-2458-13-69 PMC3559281

[CIT0017] January, A., Zebracki, K., Chlan, K.M. & Vogel, L.C., 2014, ‘Mental health and risk of secondary medical complications in adults with pediatric-onset spinal cord injury’, *Topics in Spinal Cord Injury Rehabilitation* 20(1), 1–12. 10.1310/sci2001-1 24574817 PMC3919689

[CIT0018] Jensen, M.P., Truitt, A.R., Schomer, K.G., Yorkston, K.M., Baylor, C. & Molton, I.R., 2013, ‘Frequency and age effects of secondary health conditions in individuals with spinal cord injury: A scoping review’, *Spinal Cord* 51(12), 882–892. 10.1038/sc.2013.112 24126851

[CIT0019] Jörgensen, S., Ginis, K.A., Iwarsson, S. & Lexell, J., 2017a, ‘Depressive symptoms among older adults with long-term spinal cord injury: Associations with secondary health conditions, sense of coherence, coping strategies and physical activity’, *Journal of Rehabilitation Medicine* 49(8), 644–651. 10.2340/16501977-2259 28762446

[CIT0020] Jörgensen, S., Iwarsson, S. & Lexell, J., 2017b, ‘Secondary health conditions, activity limitations, and life satisfaction in older adults with long-term spinal cord injury’, *PM and R* 9(4), 356–366. 10.1016/j.pmrj.2016.09.004 27647215

[CIT0021] Joseph, C., Delcarme, A., Vlok, I., Wahman, K., Phillips, J. & Wikmar, L., 2015, ‘Incidence and aetiology of traumatic spinal cord injury in Cape Town, South Africa: A prospective, population-based study’, *Spinal Cord* 53(9), 692–696. 10.1038/sc.2015.51 25823800

[CIT0022] Joseph, C. & Wikmar, L., 2016, ‘Prevalence of secondary medical complications and risk factors for pressure ulcers after traumatic spinal cord injury during acute care in South Africa’, *Spinal Cord* 54(7), 535–539. 10.1038/sc.2015.189 26481710

[CIT0023] Keefe, F.J. & Gil, K.M., 1986, ‘Behavioral concepts in the analysis of chronic pain syndromes’, 54(6), 776–783. 10.1037//0022-006x.54.6.776 3794021

[CIT0024] Kleinman, A., Eisenberg, L. & Good, B., 1978, ‘Culture, illness, and care. Clinical lessons from anthropologic and cross-cultural research’, *Annals of Internal Medicine* 88(2), 251–258. 10.7326/0003-4819-88-2-251 626456

[CIT0025] Lindberg, J., Kreuter, M., Taft, C. & Person, L.-O., 2013, ‘Patient participation in care and rehabilitation from the perspective of patients with spinal cord injury’, *Spinal Cord* 51(11) 834–837. 10.1038/sc.2013.97 23999110

[CIT0026] Madasa, V., Boggenpoel, B., Phillips, J. & Joseph, C., 2020, ‘Mortality and secondary complications four years after traumatic spinal cord injury in Cape Town, South Africa’, *Spinal Cord Series and Cases* 6(1), Article ID 84. 10.1038/s41394-020-00334-w PMC7473856 32887870

[CIT0027] Mashola, M.K. & Mothabeng, D.J., 2019, ‘Associations between health behaviour, secondary health conditions and quality of life in people with spinal cord injury’, *African Journal of Disability* 8, 1–9. 10.4102/ajod.v8i0.463 PMC6620481 31309047

[CIT0028] Mashola, M.K., Olorunju, S.A.S. & Mothabeng, J., 2019, ‘Factors related to hospital readmissions in people with spinal cord injury in South Africa’, *South African Medical Journal* 109(2), 107–111. 10.7196/SAMJ.2019.v109i2.13344 30834861

[CIT0029] Mayosi, B.M. & Benatar, S.R., 2014, ‘Special report: Health and health care in South Africa – 20 years after Mandela’, *The New England Journal of Medicine* 371(14), 1344–1353. 10.1056/NEJMsr1405012 25265493

[CIT0030] McKay, W.B., Sweatman, W.M. & Field-Fote, E.C., 2018, ‘The experience of spasticity after spinal cord injury: Perceived characteristics and impact on daily life’, *Spinal Cord* 56(5), 478–486. 10.1038/s41393-017-0038-y 29339776

[CIT0031] Mittmann, N., Hitzig, S.L. & Craven, B.C., 2014, ‘Predicting health preference in chronic spinal cord injury’, *Journal of Spinal Cord Medicine* 37(5), 548–555. 10.1179/2045772314Y.0000000249 PMC4166189 25055719

[CIT0032] Müller, R., Landmann, G., Béchir, M., Hinrichs, T., Arnet, U., Jordan, X. et al., 2017, ‘Chronic pain, depression and quality of life in individuals with spinal cord injury: Mediating role of participation’, *Journal of Rehabilitation Medicine* 49(6), 489–496. 10.2340/16501977-2241 28597908

[CIT0033] Neubauer, B.E., Witkop, C.T. & Varpio, L., 2019, ‘How phenomenology can help us learn from the experiences of others’, *Perspectives on Medical Education* 8(2), 90–97. 10.1007/s40037-019-0509-2 30953335 PMC6468135

[CIT0034] Nevedal, A., Kratz, A.L. & Tate, D.G., 2016, ‘Women’s experiences of living with neurogenic bladder and bowel after spinal cord injury: Life controlled by bladder and bowel’, *Disability and Rehabilitation* 38(6), 573–581. 10.3109/09638288.2015.1049378 26017362

[CIT0035] Park, S.E., Elliott, S., Noonan, V.K., Thorogood, N.P., Fallah, N., Aludino, A. et al., 2017, ‘Impact of bladder, bowel and sexual dysfunction on health status of people with thoracolumbar spinal cord injuries living in the community’, *The Journal of Spinal Cord Medicine* 40(5), 548–559. 10.1080/10790268.2016.1213554 27576584 PMC5815154

[CIT0036] Piatt, J.A., Nagata, S., Zahl, M., Li, J. & Rosenbluth, J.P., 2016, ‘Problematic secondary health conditions among adults with spinal cord injury and its impact on social participation and daily life’, *The Journal of Spinal Cord Medicine* 39(6), 693–698. 10.1080/10790268.2015.1123845 26833021 PMC5137571

[CIT0037] Rimmer, J.H., Chen, M.-D. & Hsieh, K., 2011, ‘A conceptual model for identifying, preventing, and managing secondary conditions in people with disabilities’, *Physical Therapy* 91(12), 1728–1739. https://doi.rg/10.2522/ptj.20100410 22003160 10.2522/ptj.20100410

[CIT0038] Robinson-Whelen, S., Taylor, H.B., Hughes, R.B., Wenzel, L. & Nosek, M.A., 2014, ‘Depression and depression treatment in women with spinal cord injury’, *Topics in Spinal Cord Injury Rehabilitation* 20(1), 23–31. 10.1310/sci2001-23 24574819 PMC3919691

[CIT0039] Rohn, E.J., Nevedal, A.L. & Tate, D.G., 2020, ‘Narratives of long-term resilience: Two cases of women aging with spinal cord injury’, *Spinal Cord Series and Cases* 6(1), 23. 10.1038/s41394-020-0267-8 32303683 PMC7165166

[CIT0040] Sothmann, J., Stander, J., Kruger, N. & Dunn, R., 2015, ‘Epidemiology of acute spinal cord injuries in the Groote Schuur hospital acute spinal cord injury (GSH ASCI) unit, Cape Town, South Africa, over the past 11 years’, *South African Medical Journal* 105(10), 835–839. 10.7196/SAMJnew.8072 26428588

[CIT0041] Thanh, N.C., Thi, T. & Thanh, L., 2015, ‘The interconnection between interpretivist paradigm and qualitative methods in education’, *American Journal of Educational Science* 1(2), 24–27, viewed 13 August 2020, from https://pdfs.semanticscholar.org/79e6/888e672cf2acf8afe2ec21fd42a29b2cbd90.pdf.

[CIT0042] Turk, D.C., Fillingim, R.B., Ohrbach, R. & Patel, K.V., 2016, ‘Assessment of psychosocial and functional impact of chronic pain’, *Journal of Pain* 17(9), T21–T49. 10.1016/j.jpain.2016.02.006 27586830

[CIT0043] Wahman, K., Wikmar, L.N., Chlaidze, G. & Joseph, C., 2019, ‘Secondary medical complications after traumatic spinal cord injury in Stockholm, Sweden: Towards developing prevention strategies’, *Journal of Rehabilitation Medicine* 51(7), 513–517. 10.2340/16501977-2568 31243468

[CIT0044] World Health Organization, 2004, *Promoting mental health*, World Health Organization, Genenva. 10.5840/ncbq201616462

[CIT0045] World Health Organization, 2013, *How to use the ICF: A practical manual*, viewed 17 April 2020, from https://www.who.int/classificatio0ns/drafticfpracticalmanual2.pdf?ua=1.

[CIT0046] Yorkston, K.M., Johnson, K., Boesflug, E., Skala, J. & Amtmann, D., 2010, ‘Communicating about the experience of pain and fatigue in disability’, *Quality Life Research* 19(2), 243–251. 10.1007/s11136-009-9572-1 PMC2844855 20033786

